# Modern Sociology

**Published:** 1899-09-02

**Authors:** 


### Modern Sociology.

### NERVE-TRAINING.

In an American newspaper we lately read the following story: A lady, calling upon a friend one morning, found her in a terrible state of worry. The cause of this was that some guests, whom she had expected to arrive at one o'clock, had telegraphed that they would come at twelve. "And I have so much to do! I can never be ready for them in time!" The visitor pulled off her gloves, and asked if she could be of any service. "Can I help you to prepare your guest-chamber?" "Do you think I left that till to-day?" was the indignant reply. "It is quite ready." "Then can I do some marketing for you, or help to prepare some of the dishes for luncheon?" But there was no need for that. "I should think very little of myself if I hadn't everything in the house before this, and my cook has the meal well advanced." "I can help you to set the table, then, or arrange the flowers?" But even this was all done, as the hostess proudly assured her friend; and, on prosecuting the inquiry further, it transpired that the only thing this over-burdened woman had to do before her guests arrived was to mix some mustard. "She never liked to do that before the last moment; mustard got stale so soon." From that time, says the narrator, "mustard to mix" became in the visitor's household a synonym for needless fret and worry.

There is no doubt that many of us get into the mental condition of the unhappy hostess in the anecdote. It springs from overwork and anxiety already undergone, and has for its symptom the feeling that there is more work and worry still to undergo. There are, indeed, some cases where the nervous strain is simply the result of a foolish habit, indulged instead of being restrained, because those who suffer from it think it a virtuous habit instead of a vicious one. Mrs. A. wonders how Mrs. B. can take things so lightly. As for herself, what with her anxiety about the children, and the worry of servants, and the blundering—if it is not absolute dishonesty—of the tradespeople, she has no rest from morning to night, and is wearing herself to a thread-paper! Yet, if she would only use her eyes, she would see that Mrs. B.'s house is as well kept as her own, Mrs. B.'s husband and children as healthy as hers, and ever so much happier for not having a wife and mother who is always fretful and cross. Or C. has grave doubts of D.'s solvency, because he goes out and plays golf on Saturday afternoons. Why, he (C.) did not leave the office till near midnight on Saturday, and took some work home to do on Sunday. Now, when a man or woman comes to the condition of making a boast of his or her propensity to worry, he or she is almost past cure. There is no fault so incorrigible as that which is mistaken for a virtue.

But many people know that such a condition is an unhealthy one, that there is no reason for all their anxiety, and nothing to be gained by it, and yet are unable to control their feeling of worry, with its concomitants of insomnia and ill-health. Sometimes, indeed, they do try to control it by stimulants or narcotics, and it is not difficult to predict what the end of that must be. For such treatment does not strengthen the nerves, but weakens them still further. Yet there is no doubt that many people who know all this seek to keep the nervous symptoms under by such unhealthy means because they know of no proper treatment. Their symptoms are so vague that they shrink from going to their doctor. He might only laugh at them, or he might, if he took them seriously, regard them as more or less mad, and suggest a prolonged stay in some retreat. Now, there is nothing that a nervous person dreads more than being looked upon as a "mental case," and it must be confessed that the prospect of being shut up with a number of other melancholiacs is by no means a cheering one. What they want is some treatment which shall not, if possible, necessitate their leaving home, and thus betraying to all their acquaintances that they are out of sorts; above all, some treatment which they can apply to themselves.

These remarks are suggested by some lectures on nerve-training recently delivered, the intention of which was to give such information to nervous sufferers as would help them, if not too weak-willed, to overcome their infirmity. They were taught to refrain from overwork, to economise their strength, to compel themselves to rest, and only to analyse their emotions to the extent of asking themselves, when things seem to go wrong and everyone seems inconsiderate and exacting, if it is they themselves, or the other persons or inanimate things, which are out of gear. Of course, this is not all. Mere lecturing on the duty of keeping one's temper we may all get from any friend or relation who has no pretensions to being a nerve-trainer. There are rules as to rest and exercise to be followed. So many persons do not know how to rest! One is not inevitably resting because one sits in an easy-chair, or even lies in bed, if every sense is kept on the alert to hear the slightest sound that can disturb, or if there is no determined effort to keep away all troublous thoughts. "But it's my nerves," the patient may say; "I can't control it." But one can, to a certain extent, control one's muscles, and, if this be done, it is wonderful how the nerves will follow suit. To lie down, fully stretched out, with the eyes closed and the muscles at the back of the neck relaxed will soothe many anxieties—that is, will prevent the body from being kept on the strain by troubles, real or imaginary, which belong to the mind. We all know how things that look gloomy in the extreme at night may take an aspect much less dark in the morning, when sleep has refreshed both mind and body. What we want to learn is how to obtain something like the same refreshment in the daytime, and even without the unconsciousness of sleep. Sleep will follow in due course. Sleep, like some even more desirable things, cometh not with observation. The victim of insomnia keeps away the rest he craves for by his very anxiety about it. The first thing is to put away all thought of the matter. In fact, the ideal at which the nervous sufferer ought to aim is a sort of Nirvana, or, more precisely, the condition of the youth described by Dibdin, who "walked along thinking of nothing at all." That young man had no nerves, or, rather, his nerves were in such admirable order that, like well-trained servants, they did not obtrude their presence upon him. In the rush of our modern life it may not always be easy to attain such an ideal state; but a conscientiously-followed course of nerve-training may help us at least not to be slaves, but masters of our nerves.

